# Isolation and analysis of a very virulent Marek’s disease virus strain in China

**DOI:** 10.1186/1743-422X-10-155

**Published:** 2013-05-20

**Authors:** Zhenhua Gong, Lijuan Zhang, Jianlin Wang, Linlin Chen, Hu Shan, Zhiliang Wang, Hongchao Ma

**Affiliations:** 1 China Animal Healthy and Epidemic Center, Qingdao 266032, China; 2 Qingdao Agriculture University, Qingdao 266109, China

**Keywords:** Marek’s disease virus, Isolation, PCR, Oncogenic gene, Virulence, Immunization

## Abstract

**Background:**

A severe MD was broken out at a farm in Shandong, China, despite FC126 vaccination of the chickens at 1-day-old. The mortality of the flocks reached up to 38.3%. The infected chickens were found to have MD pathological changes, including enlargement of spleens, livers and kidneys, and tumors occured on organs later. Samples were collected from the chickens for diagnosis.

**Methods:**

The collected samples were inoculated into primary duck embryo fibroblast (DEF) cells, and the MDV strain named SD2012-1 was isolated. In order to identify the isolate, amplification by PCR and sequencing of oncogenic Meq and vIL-8 gene were processed, the obtained sequences were compared with the sequences of reference strains, and SD2012-1 was used to challenge immunized SPF chickens.

**Results:**

A very virulent MDV isolate strain, SD2012-1, was isolated from a chicken flock in Shandong Province, China, the isolate had the characteristics of very virulent MDV-1, nucleotide and deduced amino acid sequence comparisons of Meq and vIL-8 gene of SD2012-1 with those of reference strains showed SD2012-1 had high homology with MDV strains isolated from China, SD2012-1 could break through the protection provided by HVT vaccine and HVT + SB-1 vaccine immunization and caused the mortality of SPF chickens over 60%. The immune failure occured at the farm could be due to the improper selection of vaccines. SD2012-1 produced death later and the gross postmortem lesions of chickens died early and later were different.

**Conclusions:**

MDV strain SD2012-1 isolated from Shandong Province, China was found to have the characteristics of very virulent MDV-1, which could break through the protection provided by HVT vaccine and HVT + SB-1 vaccine, the virus seemed to have a long latent period, and cause different gross postmortem lesions of chickens between chickens died early and later. A better immunization way should be chosen to prevent infection of this MDV strain in field.

## 

Marek’s Disease Virus (MDV) was an oncogenic poultry herpesvirus, causing not only death of chickens directly, but also immunosuppression of infected chickens sensitive to other pathogens, MDV strains (MDVs) had become increasingly virulent since the 1960s [1,2]. MDVs were classified into three serotypes which had major differences in genome and biological features. Serotype 1 MDVs included all the oncogenic strains and their attenuated forms; serotype 2 MDVs were non-oncogenic viruses isolated in chickens; serotype 3 MDVs were non-oncogenic viruses isolated in turkey, generally known as herpesvirus of turkey or HVT [3]. serotype 1 MDVs could be further classified into four pathotypes, including mild (m), virulent (v), very virulent (vv) and very virulent plus (vv+) strains [4,5].

Control of Marek’s disease was predominantly via vaccination of chickens, three types of vaccine had been developed for use against MD. These were herpesvirus of turkey (HVT), non-pathogenic serotype 2 MDV and non-pathogenic serotype 1. In 1970s, HVT vaccine was mainly used to control the disease. In mid-1980s, serotype 2 vaccine such as strain SB-1 was used in combination with HVT against the enhanced virulence strains. With further increase in virulence of field viruses [6], serotype 1 vaccine CVI988/Rispens [6,7] was introduced for widespread use in the 1990s. Recently, the failure of CVI988/Rispens vaccination had been reported, when used either alone or in combination with serotype 2 and/or serotype 3 vaccines, suggesting the emergence of high virulent strains [8,9].

In recent years, there were virulent MDVs circulating in China, the mortality rate of MD could reach up to 15%-60%. Isolate of MDVs in China were reported in breeder or layer flocks which had been vaccinated by HVT or CVI988/Rispens [10-12], the isolates were high pathogenic for chickens, the emergence of MDVs of increasing virulence was a significant problem for the poultry industry.

## Methods

### Samples collection

A severe MD was broken out at a farm with 3000 chickens in Shandong, China, despite FC126 vaccination of the chickens at 1-day-old. The clinic symptoms and death of MD in the chicken flocks began at about 60-day-old and continued until 135-day-old, the mortality of the flocks reached up to 38.3%. The infected chickens were found to have MD pathological changes, including enlargement of spleens, livers and kidneys, and tumors occured on organs later. Heparinised blood samples were collected from chickens with tumor lesions for diagnosis.

This experimental research on animals were performed with the approval of Experimental Animal Administrative Center of Shandong Province.

### Virus isolation

The collected samples were kept under refrigeration and transported, lymphocytes separated from the blood samples by lymphocyte separation medium were inoculated into primary duck embryo fibroblast (DEF) cells prepared from 11-day-old embryonated eggs, the inoculated DEF cells were incubated at 37°C with 5% CO2 for five days. After 3 passages, typical cytopathic effect (CPE) of the inoculated DEF cells stabilized at 96 hrs after inoculation. The MDV strain named SD2012-1 was obtained by isolation, and the DEF cell cultures of SD2012-1 were collected and stored at-196°C in 10% dimethyl sulphoxide.

### PCR amplification of oncogenic genes

The DNA of SD2012-1 as PCR templates was extracted from inoculated DEF culture samples and tissue samples with tumor lesions using mini-spin column chromatography method. In brief, samples was treated using proteolytic enzyme, then an equal volume of 100% ethanol was added to the lysate and the DNA was purified by mni-spin column chromatography. Contaminating proteins were removed via ethanol/salt washes, and the DNA was eluted for PCR.

The MDV target genes of PCR were Meq and vIL-8 gene, which were reported to have the greatest possibility being associated with viral oncogenicity and pathogenicity [1]. Primers for Meq and vIL-8 gene had been used to amplify the same genes of 18 MDV isolates from China [11], the PCR product of Meq and vIL-8 gene of SD2012-1 were produced individually by amplification [11] of the virus DNA extracted from DEF cultures and tissue samples infected with SD2012-1.

In order to verify the absence of infection of chickens with avian leukosis virus (ALV) and reticuloendotheliosis virus (REV), all the samples for amplification of MDV Meq and vIL-8 genes were also detected by previously reported PCR method for ALV [13] and REV [14].

The primers which had been used for PCR amplification of MDV, ALV and REV were shown in Table 1.

**Table 1:** Primers for MDV Meq and vIL-8 genes, for ALV and REV

**Virus/Gene**	**Primer sequence**		**DNA sizes**
Meq	F: 5’-GGCACGGTACAGGTGTAAAGAG-3’	R: 5’-GCATAGACGATGTGCTGCTGAG-3’	1081 bp
vIL-8	F: 5’-GAGACCCAATAACAGGGAAATC-3’	R: 5’ -TAGACCGTATCCGTGCTCCATC-3’	886 bp
ALV	F: 5’-AATTCTGCTTGAAATATG-3’	R: 5’-AGTTGTCAGGGAATCGA-3’	436 bp
REV	F: 5’-CATACTGGAGCCAATGGTGTAAAGGGCAGA-3’	R: 5’-AATGTTGTAGCGAAGTACT-3’	291 bp

### DNA cloning and sequencing of SD2012-1 strain

The PCR products of Meq gene and vIL-8 gene of SD2012-1, corresponding to the predicted size, were purified from agarose gel, and were ligated with a TA cloning vector pMD19-T, and then were transformed into DH5a competent cell individually. The cloned Meq and vIL-8 genes were then sequenced by Sanger dideoxy sequencing method.

### Comparison of Meq and vIL-8 gene of SD2012 with reference strains

The obtained Meq and vIL-8 nucleotide sequence and the deduced amino acid sequence of SD2012-1 were compared with 20 reference MDVs for the homology analysis with the use of MegAlign program. Among these 20 reference strains, 3 strains were vaccine strains, 11 strains were isolated from China, and 6 strains were isolated from USA. 20 strains were used for comparison of Meq genes, and 8 strains were used for comparison of vIL-8 genes. The MDV reference strains were retrieved from the GenBank database, and the backgrounds of the reference strains used in this study were listed in Table 2.

**Table 2:** MDV reference strains published in GenBank

**MDV strains**	**Virulence**	**Geographic origin**	**Year of isolation**	**Accession number**
0093	High virulence	Guangxi, China	2002	AF493550(M)
0095	High virulence	China	2002	AF493552(M)
0297	High virulence	China	2002	AF493553(M)
0304	High virulence	Guangxi, China	2002	AF493554(M)
G2	High virulence	Guangxi, China	2002	AF493556(M)
GX070060	High virulence	China	2008	EU427303(M)
GX070079	High virulence	China	2008	EU427304(M)
GXY2	High virulence	China	2007	EF546430(M)
YLO40920	High virulence	China	2005	DQ174459(M)
LS	High virulence	Sichuan, China	2008	HQ638149(M, V)
LMS	High virulence	Sichuan, China	2007	HQ658622(M, V)
3004	Vaccine strain	Russia	N/A	EU032468(M)
814	Vaccine strain	China	N/A	GU354326(M)
CVI988	Vaccine strain	Netherland	1972	DQ534538(M, V)
CU-2	Mild virulence	USA	N/A	EU499381(M, V)
GA	virulence	USA	1964	AF147806(M,), AF065430(V)
Md5	Very virulenc e	USA	1979	AF243438(M, V)
RB1B	Very virulence	USA	1982	EF523390(M, V)
648A	Very virulence plus	USA	1997	AY362725(M), DQ534534(V)
584A	Very virulence plus	USA	Before 2000	DQ534532(M, V)

“M” refers to accession number of Meq gene; “V” refers to accession number of vIL-8 gene; “N/A” refers to data not available.

### Virulence studies of SD2012-1

90 SPF chickens randomly divided into three groups were vaccinated at the age of one day, In group 1, 30 chickens were vaccinated with 2000 PFU HVT (FC126 strain, 2000 PFU HVT had been the suggested dosage used at the farm with MD broken out); in group 2, 30 chickens were vaccinated with bivalent vaccine (2000 PFU FC126 + 1200 PFU SB-1); in group 3, 30 chickens were inoculated with phosphate buffer. At the age of ten days, all the chickens were challenged intra-abdominally with 0.5 ml diluent of plaque purified SD2012-1 strain containing 4000 PFU. Clinical signs and gross postmortem lesions were recorded after virus challenge. Organs of chickens with lesions were sampled for PCR diagnosis.

## Results

### PCR amplification for Meq and vIL-8 gene of SD2012-1

**Figure 1:**
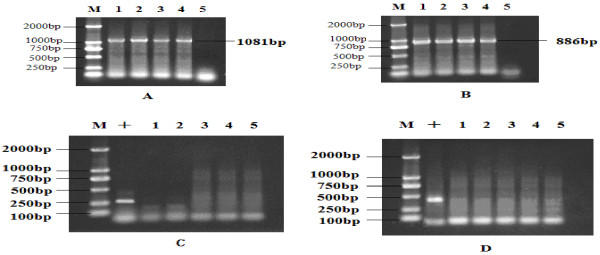
**Detection of MDV, ALV and REV by PCR.** (**A**) PCR amplification of Meq gene; (**B**) PCR amplification of Vil-8 gene. (**C**) Detection of ALV by PCR; (**D**) Detection of REV by PCR; (M) DL 2000 DNA Marker; (+) Positive control of ALV in (**C**) or REV in (**D**). (1) Spleen of infected chicken from farm with MD broken out; (2) DEF culture infected with MDV SD_2012-1_; (3) Spleen of 55-day-old died chicken without tumor; (4) Spleen of 90-day-old chicken with tumor; (5) DEF culture without infection.

By PCR [11] of the extracted virus DNA with primers in Table 1, the specific presence of an approximately 1081 bp long DNA product for MDV Meq gene (Figure 1-A) and the specific presence of an approximately 886 bp long DNA product for MDV vIL-8 gene (Figure 1-B) were found in samples, including the DEF cultures infected with SD2012-1 isolate, samples collected from chickens infected with SD2012-1 at the farm and samples collected from challenged chickens with SD2012-1, the PCR products from all the samples were obvious, which verified the existence of infection with MDV to chickens and inoculated DEF cells.

### Detection of ALV and REV by PCR

All the samples, including the DEF cultures infected with SD2012-1 isolate, samples collected from chickens infected with SD2012-1 at the farm and samples collected from challenged chickens with SD2012-1were verified to be negative of Avian Leukosis virus (Figure 1-C) and Reticuloendotheliosis virus (Figure 1-D) by PCR, which showed the absence of ALV and REV in these samples.

### sequence of Meq and vIL-8 gene of SD2012-1

By sequencing, the nucleotide sequence of Meq and vIL-8 gene of SD2012-1 and their deduced amino acid sequence were obtained, the sequences of SD2012-1 were submitted to the GenBank database with the accession number KC511815 for Meq gene and KC511812 for vIL-8 gene.

### Sequence analysis of SD2012-1

Comparisons of Meq and vIL-8 gene of SD2012-1 with those of reference strains showed SD2012-1 had high homology with the reference strains.

Comparisons of the nucleotide sequence of Meq gene of SD2012-1 with the sequence of 20 reference strains in Table 2 showed the nucleotide homology of SD2012-1 isolate with the 20 strains was between 94.7-99.8%, SD2012-1 had the highest homology with LS at 99.8%, and had the lowest homology with CVI988 at 94.7%.

**Figure 2:**
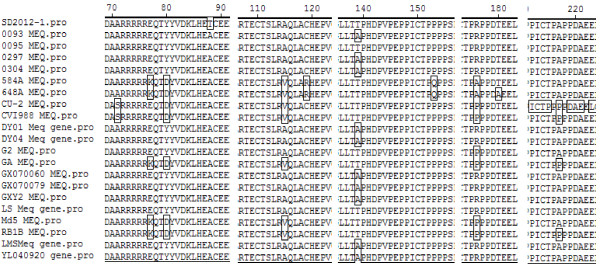
Alignment for amino acid mutation site on Meq gene of 21 MDVs.

The Meq amino acid of SD2012-1 was 339aa long, comparisons of Meq protein amino acid sequence of SD2012-1 with the sequence of 20 strains in Table 2 showed the amino acid homology of SD2012-1 isolate compared with the 20 strains was between 96.5-99.4%, SD2012-1 had the highest amino acid homology with LS at 99.4%, and had the lowest homology with CVI988 at 96.5%. As most strains isolated in China, SD2012-1 had the same amino acid mutation of Meq protein (Figure 2) at position 71(S to A), 77(K to E), 80(D to Y), 115(V to A), 119(R to C), 153(Q to P), 176(P to R) and 217(P to A ), except no mutation of SD2012-1 at position 139(remain T).

Comparison of the nucleotide sequence of vIL-8 gene of SD2012-1 with the sequence of 8 strains, CVI988, CU-2, GA, RB1B, 648A, 584A, LS, LMS, showed the nucleotide homology of SD2012-1 isolate with 8 reference strains were between 99.4-99.6%, SD2012-1 had the highest homology with LS, RB1B and CU-2 at 99.6%, and had the lowest homology with CVI988 and 584A at 99.4%.

**Figure 3:**
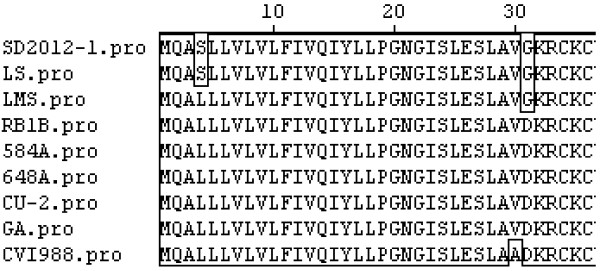
Alignment for amino acid mutation site on vIL-8 gene of 9 MDVs.

The amino acid of SD2012-1 was 134aa long, comparison of vIL-8 protein amino acid sequence of SD2012-1 with CVI988, CU-2, GA, RB1B, 648A, 584A, LS and LMS showed that the amino acid homology of SD2012-1 isolate with 8 strains was between 97.8-99.3%, SD2012-1 had the highest homology with LS at 99.3%, and had the lowest homology with CVI988 at 97.8%. The vIL-8 gene amino acid sequence of CU-2, GA, RB1B, 648A, 584A were conservative without amino acid mutation between the 5 strains. As LS did, SD2012-1 had the same amino acid mutation of vIL-8 protein (Figure 3) at position 4(L to S) and 31(D to G) compared with CU-2, GA, RB1B, 648A, 584A .

### Virulence of SD2012-1

**Figure 4:**
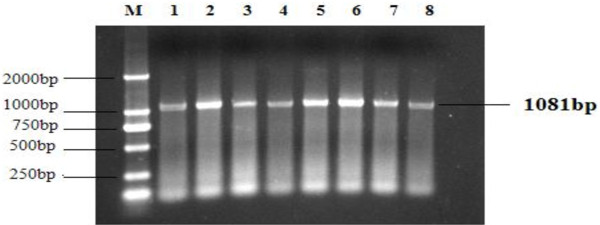
**PCR amplification of Meq gene from tissues of chickens infected with SD**_**2012-1**_**.** (1) Feather pulp; (2 ) liver; (3) kidney; (4) muscular stomach; (5) mesentery; (6) duodenum; (7) heart; (8) bursa of Fabrius.

**Figure 5:**
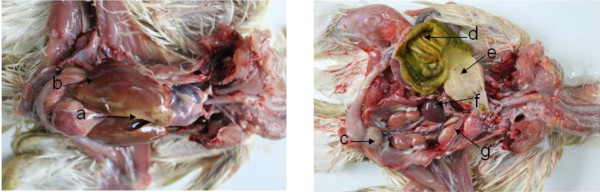
**Pathological changes of chickens died at 55-day-old. **(**a**) yellow degeneration diffusely in liver edge; (**b**) rough and uneven in liver surface; (**c**) bursa of Fabricius swelling; (**d**) muscular stomach swelling; (**e**) glandular stomach swelling; (**f**) lots of white diffused nodule in the swelled spleen; (**g**) kidney swelling and pale in color.

**Figure 6:**
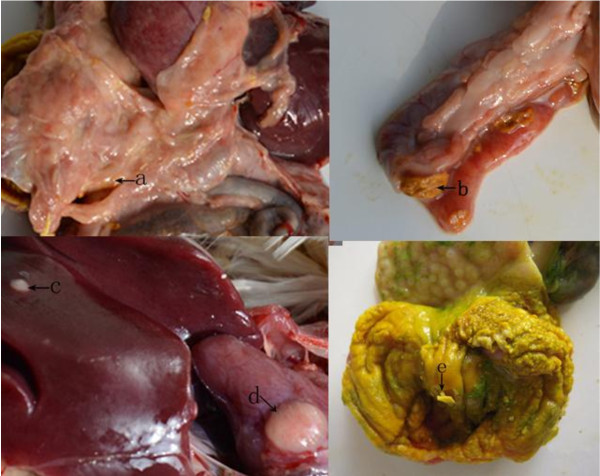
**Pathological changes of chickens died at 90-day-old. a**) diffused nodule in the thicken mesentery; (**b**) ulcer in duodenal mucosa; (**c**) tumor in liver; (**d**) tumor in heart; (**e**) muscular stomach swelling with thicken corneum layer, and easy to be peeled.

No clinical sign was observed during the first 6 week post challenge of SD2012-1. The earliest death appeared on 45 days post challenge in no vaccine group, and on 48 days post challenge in HVT immunized group and FC126 + SB-1 group. The peak of death came on 60–85 days post challenge. The mortality were 70.3% in no vaccine group, 66.7% in HVT immunized group and 60% in FC126 + SB-1 immunized group (Table 3). Organic samples collected from the chickens with lesions were detected to be MDV positive by PCR (Figure 4). the main gross lesion of the early died chickens were degeneration and swelling of organs without development of tumor (Figure 5). The gross lesion of the later died chickens were mainly tumors developed on liver, heart and mesentery, and gizzard swelling with thicken corneum layer which was easy to be peeled (Figure 6).

**Table 3:** **Statistics of chickens challenged with MDV SD**_**2012-1**_**isolate**

**Group**	**Bird number**	**Death with tumor**	**Tumor lesion**	**Total (%)**
no vaccine	30	9	13	22 (70.3%)
HVT	30	8	12	20 (66.7%)
HVT + SB1	30	8	10	18 (60%)

## Discussion

In the past 40 years, the virulence of MDVs had been increasing gradually, which was possibly caused by the selective pressure of vaccination [5,15-18]. With isolation and gene sequencing, the oncogenic gene changes emerged in the predominant strains were monitored, and the effectiveness of existing vaccines were evaluated [19], both played an important role on MD prevention. In recent years, MD occurred frequently in China in spite of vaccination, a number of MDVs were isolated from different area in China, molecular epidemiologic studies on the amino acid sequences of oncogenic gene of MDVs indicated that the amino acid mutations in Meq gene and vIL-8 gene displayed perfect regularity in MDVs circulating in China, which could be considered as features of field MDVs prevalent in recent years in China [11,12,20].

In this study, a virulent MDV isolate which caused severe death of chicken at the infected farm was isolated by transfecting monolayer of duck embryo fibroblast (DEF) with the peripheral blood lymphocytes (PBLs) of the infected chicken, the MDV isolate strain named SD2012-1 was isolated from a chicken flock in Shandong Province, China, which had been vaccinated with HVT vaccine, the isolate was found to have the characteristics of virulent MDV-1.

The oncogenic Meq gene and vIL-8 gene of SD2012-1 were amplified by PCR and sequenced. Compared with 20 reference strains, SD2012-1 had the highest Meq nucleotide sequence homology with LS, a virulent strain isolated from China, at 99.8%, and had the lowest nucleotide sequence homology with CVI988 at 94.7%. As most strains isolated in China [11], the Meq amino acid of SD2012-1 was 339aa long, which was 59aa shorter than Meq protein of CU-2 and CVI988 which had a piece of ammino acid insertion resulting suppression of Meq expression [21,22]. The Meq amino acid of SD2012-1contains a basic leucine zipper (bZIP) domain at the N terminal closely resembling the jun/fos oncogene family [23], SD2012-1 had the highest Meq amino acid homology with LS at 99.4%, and had the lowest amino acid homology with CVI988 at 96.5%. Like the 11 compared reference strains from China in Table 2, SD2012-1 had the same amino acid mutation of Meq gene at position 71 (S to A), 77 (K to E), 80 (D to Y), 115 (V to A), 119 (R to C), 153 (Q to P), 176 (P to R) and 217 (P to A ), which displayed regularity of strains isolated from China [11,12], except no mutation of SD2012-1 at position 139 (remain T no A), and threonine (T) was also found in GA, Md5, RB1B, 648A, 584A strain at position 139. It was reported that amino acid change at position 77 (E to K) was the feature of high virulent MDV strains, and a glutamic acid (E) at position 77 was associated with lower virulence [24], however, SD2012-1 and the 11 compared reference strains from China had glutamic acid (E) at position 77, and all the 12 MDV strains from China were high virulence strains, this could demonstrate that a glutamic acid (E) at position 77 was not necessarily a feature of MDV strains of lower virulence. Like mild virulence, virulence and very virulence reference strains in Table 2, SD2012-1 had the amino acid of cysteine(C) at position 119, whereas an arginine(R) to C mutation at position 119 is found in US vv + MDVs 584A and the 648A strain [11].

vIL-8 gene was reported to have possibility to be associated with viral oncogenicity and pathogenicity [25], Comparing SD2012-1 with 8 reference strains, CVI988, CU-2, GA, RB1B, 648A, 584A, LS, LMS, showed SD2012-1 had the highest vIL-8 nucleotide sequence homology with LS, RB1B and CU-2 at 99.6%, and had the lowest nucleotide sequence homology with CVI988 and 584A at 99.4%. The amino acid of vIL-8 of SD2012-1 was 134aa long, the amino acid homology of SD2012-1 isolate with 8 strains was between 97.8-99.3%, SD2012-1 had the highest amino acid homology with LS at 99.3%, and had the lowest amino acid homology with CVI988 at 97.8%. The vIL-8 gene amino acid sequence of CU-2, GA, RB1B, 648A, 584A were conservative without amino acid mutation between the 5 strains. SD2012-1 had the amino acid mutation of vIL-8 gene at position 4 (L to S) and position 31 (D to G) compared with CU-2, GA, RB1B, 648A, 584A. It should be noticed that the amino acid mutations of vIL-8 of SD2012-1 at position 4 and position 31 were found in most isolates from China [11], which displayed regularity of strains isolated from China [11].

Although vaccines like HVT, HVT + SB-1 and CVI988 were widely used against Marek’s disease in China, HVT was more often only used at farms for its easier storage and usage than the others, this would result in the high risks of infection with high virulent Marek’s disease strains. The virulence study of SD2012-1 revealed that SD2012-1 characterized very virulence to SPF chickens, the isolate could break through the protection provided by HVT vaccine and HVT + SB-1 vaccine immunization and caused chicken mortality over 60%, it also revealed that the immune failure occured at the farm was due to the selection of HVT vaccination. We were taking another experimental protection trial including vaccination with CVI988 against SD2012-1, the study would be reported in another paper.

Unlike LS strain isolated in Sichuan, China, which could produced high early mortality and tumor in chicken 18 days post challenge [11], SD2012-1 seemed to have a long latent period, it produced death later on 45 days post challenge in no vaccine group, and 48 days post challenge in HVT immunized group. The peak of death came on 60–85 days post challenge.

Gross postmortem lesions were found in chickens died early with degeneration in liver, swelling of bursal and gizzard, tumors were not found in early died chickens. Although these lesions are atypical for MDV, the MDV positive detection result (Figure 4) by PCR of the organs with atypical lesion for MDV confirmed their infection with MDV. Typical MD lesion with gross tumor development was found 75 days post challenge, 30 days after the early death of MD was found, tumors could be found on liver, heart and mesentery of the later died chickens, the chickens were detected to be MDV positive by PCR.

## Conclusion

In conclusion, a very virulent MDV isolate strain named SD2012-1 was isolated from a chicken flock in Shandong Province, China, which had been vaccinated with HVT vaccine, the isolate was isolated by transfecting monolayer of duck embryo fibroblast (DEF).

SD2012-1 was found to have the characteristics of very virulent MDV-1, SD2012-1 had the highest Meq amino acid homology with LS at 99.4%, and had the lowest homology with CVI988 at 96.5%. SD2012-1 had the same amino acid mutation of Meq gene at position 71, 77, 80, 115, 119, 153, 176 and 217, which displayed regularity of strains isolated from China [11,12], except no mutation of SD2012-1 at position 139aa.

SD2012-1 had the highest vIL-8 nucleotide sequence homology with LS, RB1B and CU-2 at 99.6%, and had the lowest homology with CVI988 and 584A at 99.4%. SD2012-1 had the amino acid mutation of vIL-8 gene at position 4 (L to S) and position 31 (D to G), which displayed regularity of strains isolated from China [11].

SD2012-1 could break through the protection provided by HVT vaccine and HVT + SB-1 vaccine immunization and caused chicken mortality over 60%, and the immune failure occured at the farm could be due to the selection of HVT vaccination.

SD2012-1 seemed to have a long latent period, it produced death later on 45 days post challenge in no vaccine group, and 48 days post challenge in HVT immunized group. The gross postmortem lesions of chickens challenged by SD2012-1 died early and later were different, the main gross lesion of the early died chickens were degeneration and swelling of organs without development of tumor, and gross tumor lesion occurred in organs and tissue was found later.

A better immunization way should be chosen to prevent infection of this MDV strain in field.

## Competing interests

The authors declare that they have no competing interests.

## Author’s contributions

ZG, LZ: carried out studies design, experiment and interpretation of the data, wrote the manuscript. JW, LC: participated in experiment and interpretation of the data. HS, ZW, HM: participated in studies design and draft of the manuscript. All authors read and approved the final manuscript.

## References

[B1] Witter RL Increased virulence of Marek’s disease virus field isolates Avian Dis 1997 41 149 163 10.2307/1592455 9087332

[B2] Osterrieder N Kamil JP Schumacher D Tischer BK Trapp S. Marek’s disease virus from miasma to model Nat Rev Microbiol 2006 4 753 761 10.1038/nrmicro1382 16541136

[B3] Bulow VV Biggs PM Differentiation between strains of Marek’s disease virus and turkey herpesvirus by immunofluorescence assays Avian Pathol 1975 4 133 146 18777301 10.1080/03079457509353859

[B4] Witter RL Characteristics of Marek’s disease viruses isolated from vaccinated commercial chicken flocks: association of viral pathotype with lymphoma frequency Avian Dis 1983 27 113 132 10.2307/1590377 6303287

[B5] Witter RL Calnek BW Buscaglia C Gimeno IM Schat KA Classification of Marek’s disease viruses according to pathotype: philosophy and methodology Avian Pathol 2005 34 75 90 10.1080/03079450500059255 16191686

[B6] Rispens BH van Vloten H Mastenbroek N Maas HJ Schat KA Control of Marek’s disease in the Netherlands. I. Isolation of an avirulent Marek’s disease virus (strain CVI 988) and its use in laboratory vaccination trials Avian Dis 1972 16 108 125 10.2307/1588905 4337307

[B7] de Boer GF Groenendal JE Boerrigter HM Kok GL Pol JM Protective efficacy of Marek’s disease virus (MDV) CVI-988 CEF65 clone C against challenge infection with three very virulent MDV strains Avian Dis 1986 30 276 283 10.2307/1590529 3015113

[B8] Burgess SC Young JR Baaten BJ Hunt L Ross LN Parcells MS Kumar PM Tregaskes CA Lee LF Davison TF Marek’s disease is a natural model for lymphomas overexpressing Hodgkin ‘s disease antigen (CD30) Proc Natl Acad Sci U S A 2004 101 13879 13884 10.1073/pnas.0305789101 15356338 PMC518847

[B9] Schumacher D Tischer BK Teifke JP Wink K Osterrieder N Generation of a permanent cell line that supports efficient growth of Marek’s disease virus (MDV) by constitutive expression of MDV glycoprotein E J Gen Virol 2002 83 1987 1992 12124462 10.1099/0022-1317-83-8-1987

[B10] Chen M Payne WS Hunt H Zhang H Holmen SL Dodgson JB Inhibition of Marek’s disease virus replication by retroviral vector-based RNA interference Virology 2008 377 265 272 10.1016/j.virol.2008.03.019 18570965

[B11] Tian M Zhao Y Lin Y Zou N Liu C Liu P Cao S Wen X Huang Y Comparative analysis of oncogenic genes revealed unique evolutionary features of field Marek’s disease virus prevalent in recent years in China Virol J 2011 8 121 131 10.1186/1743-422X-8-121 21406076 PMC3068976

[B12] Zhang Y Liu C Zhang F Shi W Li J Sequence analysis of the Meq gene in the predominant Marek’s disease virus strains isolated in China during 2006–2008 Virus Genes 2011 43 353 357 10.1007/s11262-011-0645-1 21789633

[B13] Smith EJ Williams SM Fadly AM Detection of avian leukosis virus subgroup J using the polymerase chain reaction Avian Dis 1998 42 2 375 380 10.2307/1592488 9645329

[B14] Awad AM Abd El-Hamid HS Abou Rawash AA Ibrahim HH Detection of reticuloendotheliosis virus as a contaminant of fowl pox vaccines Poult Sci 2010 89 2389 2395 10.3382/ps.2010-00899 20952701

[B15] Kross I Davis PJ Shilleto RW Isolation of highly cytolytic MDV strains from Germany and Spain Avian Pathol 1998 27 313 315 10.1080/03079459808419343 18484005

[B16] Sung HW High virus titer in feather pulp of chickens infected with subgroup J avian leukosis virus Avian Dis 2002 46 517 524 10.1637/0005-2086(2002)046[0517:RIOMSD]2.0.CO;2 12061636

[B17] Dudnikova E Norkina S Vlasov A Slobodchuk A Lee LF Witter RL Evaluation of Marek’s disease field isolates by the “best fit” pathotyping assay Avian Pathol 2007 36 135 143 10.1080/03079450701209857 17479374

[B18] Buscaglia C Nervi P Risso M Characterization of four very virulent Argentinian strains of Marek’s disease virus and the influence of one of those isolates on synergism between Marek’s disease vaccine viruses Avian Pathol 2004 33 190 195 10.1080/03079450310001652103 15276986

[B19] Tan J Cooke J Clarke N Tannock GA Optimization of methods for the isolation of Marek’s disease viruses in primary chicken cell cultures J Virol Methods 2008 147 312 318 10.1016/j.jviromet.2007.09.011 17976832

[B20] Teng L Wei P Song Z He J Cui Z Molecular epidemiological investigation of Marek’s disease virus from Guangxi, China Arch Virol 2011 156 203 206 10.1007/s00705-010-0840-8 21053030

[B21] Chang K Ohashi K Onuma M Diversity (polymorphism) of the Meq gene in the attenuated Marek ‘s disease virus (MDV) serotype 1 and MDV-transformed cell lines J Vet Med Sci 2002 64 1097 1101 10.1292/jvms.64.1097 12520100

[B22] Lee LF Wu P Sui D Ren D Kamil J Kung HJ Witter RL The complete unique long sequence and the overall genomic organization of the GA strain of Marek’s disease virus Proc Natl Acad Sci U S A 2000 97 6091 6096 10.1073/pnas.97.11.6091 10823954 PMC18563

[B23] Jones D Lee L Liu J Kung HJ Tillotson JK Marek’s disease virus encodes a basic-leucine zipper gene resembling the fos/jun oncogenes that is highly expressed inlymphoblastoid tumors Proc Natl Acad Sci U S A 1992 89 4042 4046 10.1073/pnas.89.9.4042 1315048 PMC525628

[B24] Shamblin CE Greene N Arumugaswami V Dienglewicz RL Parcells MS Comparative analysis of Marek’s disease virus (MDV) glycoprotein-, lytic antigen pp 38- and transformation antigen Meq-encoding genes: association of Meq mutations with MDVs of high virulence Vet Microbiol 2004 102 147 167 10.1016/j.vetmic.2004.06.007 15327791

[B25] Izumiya Y Jang H Ono M Mikami T A complete genomic DNA sequence of Marek’s disease virus type 2, strain HPRS24 Curr Top Microbiol Immunol 2001 255 191 221 10.1007/978-3-642-56863-3_8 11217423

